# Prevalence of Self-Reported Gluten-Related Disorders and Adherence to a Gluten-Free Diet in Salvadoran Adult Population

**DOI:** 10.3390/ijerph15040786

**Published:** 2018-04-18

**Authors:** Noé Ontiveros, Cecilia Ivonne Rodríguez-Bellegarrigue, Gerardo Galicia-Rodríguez, Marcela de Jesús Vergara-Jiménez, Elia María Zepeda-Gómez, Jesús Gilberto Arámburo-Galvez, Martina Hilda Gracia-Valenzuela, Francisco Cabrera-Chávez

**Affiliations:** 1 División de Ciencias e Ingeniería, Departamento de Ciencias Químico Biológicas y Agropecuarias, Universidad de Sonora, Navojoa, Sonora 85880, Mexico; 2 Facultad de Ciencias de la Salud Luis Edmundo Vasquez, Departamento de Salud Pública, Universidad Dr. José Matias Delgado, Antiguo Cuscatlán 1502, El Salvador; cirodriguezb@ujmd.edu.sv; 3 Nutrition Sciences Academic Unit, University of Sinaloa, Av. Cedros and Sauces Street, Los Fresnos, Culiacán, Sinaloa 80019, Mexico; gerardo.galicia.rodriguez@hotmail.com (G.G.-R.); mjvergara@uas.edu.mx (M.d.J.V.-J.); elia_zg@hotmail.com (E.M.Z.-G.); gilberto.aramburo.g@gmail.com (J.G.A.-G.); 4 Instituto Tecnológico del Valle del Yaqui, Block 611, Bácum, Valle del Yaqui, Sonora 82276, Mexico; mgracia.valenzuela@itvy.edu.mx

**Keywords:** gluten-related disorders, gluten-free diet, gluten sensitivity

## Abstract

Gluten-related disorders are not considered of relevance at public health level in Central America. The prevalence of gluten-related disorders, and adherence to a gluten-free diet, remain unknown in the Central American region. We conducted a cross-sectional survey of the Central American population from San Salvador, El Salvador, to estimate the prevalence rates of self-reported gluten-related disorders and adherence to a gluten-free diet. 1326 individuals were surveyed. Self-reported prevalence rates were (95% Confidence Interval): gluten sensitivity 3.1% (2.3–4.2); physician-diagnosed celiac disease 0.15% (0.04–0.5); wheat allergy 0.75% (0.4–1.3); non-celiac gluten sensitivity 0.98% (0.5–1.6). The prevalence rate of adherence to a gluten-free diet was 7.0% (5.7–8.5). Seven self-reported physician diagnosed gluten-sensitive cases informed the co-existence of non-celiac gluten sensitivity with celiac disease and/or wheat allergy. Among the non-self-reported gluten sensitivity individuals following a gluten-free diet, 50% reported that they were seeing a health professional for gluten-free dietary advice. Gluten sensitivity is commonly reported in Salvadoran population, but some health professionals acknowledge the coexistence of wheat allergy, celiac disease, and non-celiac gluten sensitivity. Among studies at population level, the prevalence of adherence to a gluten-free diet in Salvadoran population is the highest reported until now. However, just a few of the gluten-free diet followers were doing it for health-related benefits; the others reported weight control and the perception that the diet is healthier as the main motivation for adopting such a diet.

## 1. Introduction

The spectrum of gluten-related disorders include Wheat Allergy (WA), Celiac Disease (CD), and Non-Celiac Gluten Sensitivity (NCGS). Wheat allergy is strongly linked to anti-wheat IgE antibodies production and mast cells degranulation upon re-exposition to the allergen [1]. CD is an autoimmune-like enteropathy with a strong genetic background. Different from WA and CD, NCGS is not recognized as a strict enteropathy, and it is not linked to the specific arm of the immune system; nonetheless, innate immune mediators could play an important role in the pathogenesis of NCGS [2]. Notably, symptomatic adverse reactions are triggered after the ingestion of wheat in the three conditions, but CD is asymptomatic in many cases. Thus, WA patients should avoid wheat from their diets, but CD and NCGS cases should follow a strict gluten-free diet (GFD) to avoid the symptoms associated to these conditions and/or long-term complications in CD cases.

Algorithms for the diagnosis of WA and CD have been published and accepted by both the scientific community and healthcare professionals [3,4]. The gold standard for oral WA diagnosis is the double-blind placebo-controlled oral challenge (ingestion of wheat), followed by the evaluation of symptoms, while formally diagnosed CD requires the patient to be on a gluten-containing diet and includes gastrointestinal endoscopy with biopsies and further histological analysis (biopsy-proven CD) [5]. Due to the lack of specific biological markers, NCGS diagnosis involves the exclusion of WA and CD, followed by a double-blind placebo-controlled gluten challenge [6]. However, these protocols are not suitable for large-scale population studies, and survey-based studies have emerge as an alternative to evaluate the prevalence of gluten-related disorders at population level [7,8]. Such a survey-based protocol has allowed us to estimate the prevalence of self-reported gluten-related disorders and adherence to a GFD in three Latin American Countries [9,10,11].

Although the GFD is considered a treatment for gluten-related disorders, recent survey studies highlighted that most Latin Americans following a GFD were doing it for reasons other than health-related benefits, and without medical/dietitian advice [9,10], potentially compromising fiber and micronutrients intake [12,13,14]. On the contrary, some Latin Americans that experience recurrent adverse reactions after gluten ingestion were not following the diet. This can be attributed to the mildness of the symptoms triggered, access to gluten-free products, and/or economic aspects [9,10]; however, studies addressing these issues in Latin American populations are scarce. Furthermore, celiac disease and other gluten-related disorders are not considered of relevance at public health level in Central American Nations, and as such, no population-based study has been carried out in Central America in order to evaluate the magnitude and relevance of the problem. Certainly, the Central America population consume less wheat-based foods than other Latin American populations from North and South America (e.g., Argentina, Chile, Mexico) [15], and carries pre-Columbine genes from a native population that followed a gluten-free diet for millennia [16]. However, there has been a transition from this diet to a gluten-containing diet since the Spaniards’ introduction of wheat [16]. Thus, the aim of this study was to evaluate, at population level, the prevalence of self-reported gluten-related disorders and adherence to a gluten-free diet in a Central America population from San Salvador, El Salvador.

## 2. Materials and Methods

### 2.1. Questionnaire and Population Survey

A previously validated questionnaire was utilized for the purposes of the study [9,10,11]. The first section of the questionnaire asks about demographics and clinical characteristics and adverse reactions to oral wheat and/or gluten. The second section was designed for those that reported adverse reactions to foods other than wheat/gluten, or reported no adverse reactions to foods including gluten. Additionally, all respondents answered a question about following a GFD. Those who were following a GFD answered a question about their motivations for following the diet.

We collected data in public places from San Salvador, El Salvador, during the period 24 to 28 May 2017. Respondents were approached in urban parks and outside shopping malls and supermarkets located in San Salvador city. Inclusion criteria were as follows: (1) Salvadorian individuals; (2) ≥18 years old; and (3) subjects capable to read and answer the questionnaire by themselves. Trained nutritional sciences and medicine undergraduate students collected the data and gave assistance, when requested, related to the meaning of specific terms utilized.

### 2.2. Definitions

Individuals were classified according to previously published definitions [9], which are shown in Table 1. Convincing symptoms of wheat/food allergy were: skin with hives and angioedema, trouble breathing, wheezing or throat tightness, vomiting and diarrhea, which are considered as characteristics symptoms and signs of anaphylaxis by the World Allergy Organization [17]. To be convincing of WA, the symptoms should appear within two hours after the ingestion of wheat/food [18].

### 2.3. Statistical Analysis and Ethical Issues

The total numbers, percentages, odds ratio, and 95% confidence interval (CI) in this study were analyzed according to a set of descriptive statistics. Associations were evaluated by two-tailed Fisher’s exact test, and mean differences were calculated by Student *t*-test (*p* < 0.05) (PASW statistics version 18.0, SPSS Inc., Chicago, IL, USA). OpenEpi software version 3.03a was used to estimate the prevalence rates (95% CI). All participants signed an informed consent form when they completed the survey. The ethical review board of the University Dr. José Matías Delgado approved the study protocol (Protocol Resolution Acta 001-2017).

## 3. Results

### 3.1. Demographic and Clinical Characteristics

A total of 1326 questionnaires were considered for prevalence-rate estimations. The proportion of male:female was 50.6%:49.3%. The most common self-reported physician-diagnosed conditions were Colitis (13.95%), Lactose intolerance (12.52%) and irritable bowel syndrome (IBS) (6.26%). The risk analysis between SR-GS and non-SR-GS showed significant associations for IBS, food intolerance, colitis, and lactose intolerance (data not shown).

### 3.2. Prevalence Rates

**Figure 1:**
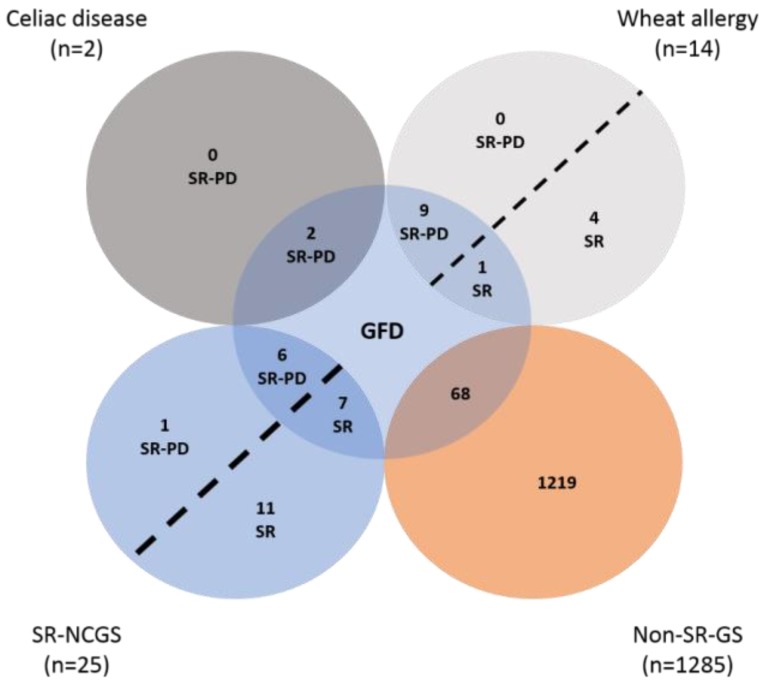
Characteristics of individuals following a GFD. Acronyms used: SR-PD: Self-Reported Physician-Diagnosed; SR: Self Reported; GFD: Gluten-free Diet; SR-NCGS: Self-Reported Non-Celiac Gluten Sensitivity; Non-SR-GS: Non Self-Reported Gluten Sensitivity.

**Figure 2:**
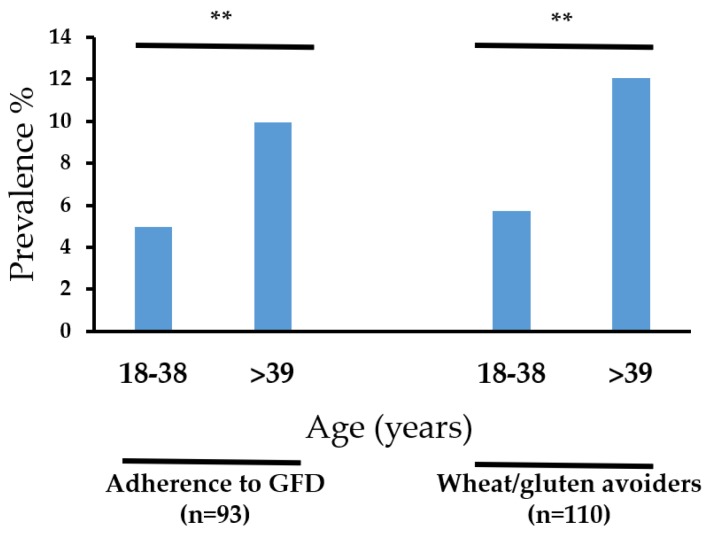
Gluten-free diet (GFD) adherence and avoiding of wheat/gluten according to age. ** *p < 0.01*.

The prevalence rates estimated are shown in Table 2. The prevalence rate of SR-GS and gluten avoiders were significantly higher in women than in men (*p* < 0.05) (Table 2). There were not significant differences by gender for all the other assessments (*p* < 0.05) (Table 2). The characteristics of the respondents following a GFD are shown in Figure 1. Notably, more than 70% (*n* = 68) of those following a GFD, and more than 80% (*n* = 92) of the gluten avoiders, were non-SR-GS cases. Further analysis showed that those aged >39 years old more commonly were following a GFD or avoiding gluten from their diets (*p* < 0.05) (Figure 2).

### 3.3. Cases with More than One Physician-Diagnosed Gluten-Related Disorder

Seven respondents reported the coexistence of NCGS, either with CD or WA, or with both conditions. Of these, four cases had to be excluded from the prevalence estimation analysis due to the uncertainty of their diagnoses. The other three cases meet criteria for WA. The characteristics of the cases are shown in Table 3.

### 3.4. Self-Reported Gastrointestinal and Extra-Intestinal Symptoms Triggered after Gluten Ingestion in SR-GS Cases

**Figure 3:**
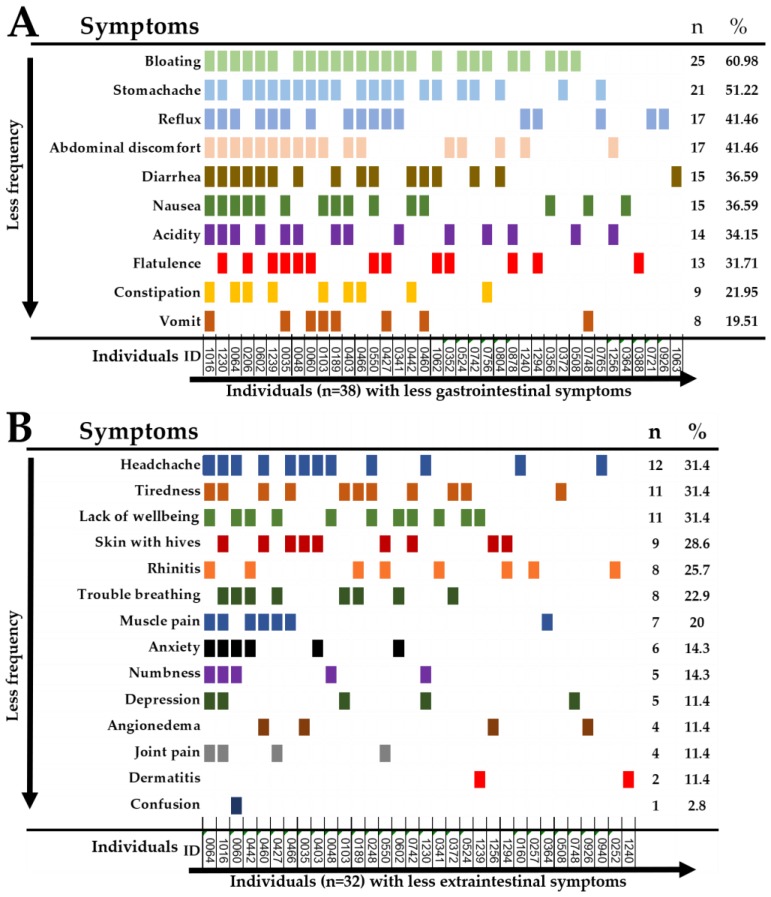
Recurrent self-reported symptoms in SR-GS individuals. (**A**); gastrointestinal symptoms, (**B**); extra-intestinal symptoms.

Forty-two individuals reported recurrent symptoms triggered after the ingestion of wheat/gluten. Ten and four individuals reported either intestinal or extraintestinal symptoms only, respectively. Bloating and stomachache were the most common recurrent gastrointestinal symptoms (Figure 3A). Headache, tiredness and a lack of wellbeing were the most common extra-intestinal symptoms (Figure 3B).

### 3.5. Reasons for Gluten-Free Dietary Non-Compliance and Motivations for Following a GFD

**Figure 4:**
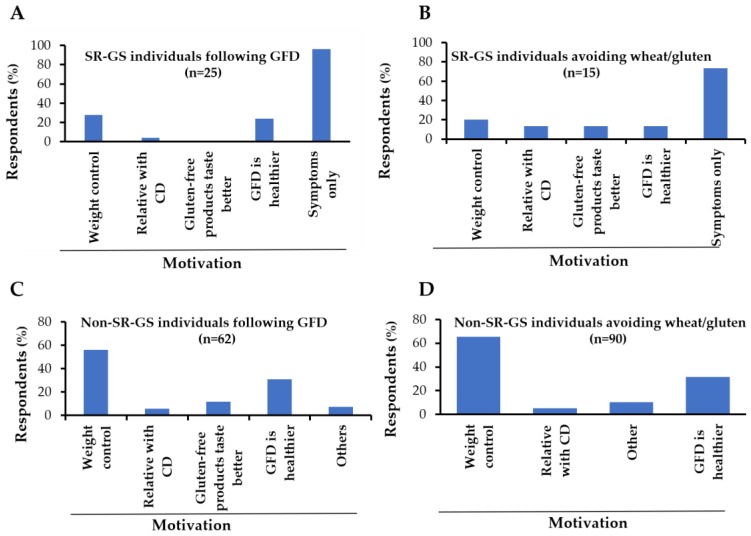
Motivations for following a gluten-free diet (GFD) or avoiding wheat/gluten from the diet. (**A**,**B**) motivations of Self-Reported Gluten Sensitivity (SR-GS) cases for following a GFD, (**C**,**D**) motivations of non Self-Reported Gluten Sensitivity (non-SR-GS) cases for avoiding wheat/gluten from their diets. Six and five non-SR-GS cases missed the question about the motivation for following a GFD or avoiding wheat/gluten from their diets, respectively.

Of those that meet criteria for SR-PD gluten-related disorders or SR-GS, 5.5% (n = 1) and 65% (n = 15) were not following a GFD respectively. The main reasons reported for gluten-free dietary non-compliance were the low availability of gluten free products (46.1%) and the high cost (30.7%), followed by the mildness of the symptoms triggered after gluten ingestion (23.1%). Regarding the motivations for following a GFD in the SR-GS group, the most common motivation reported was the symptoms triggered after wheat/gluten ingestion (Figure 4A). Similar results were obtained for the gluten avoiders group that meet criteria for SR-GS (Figure 4B). In contrast, weight control and the perception that a GFD is healthier were the main motivations for either following a GFD or avoiding wheat/gluten in the non-SR-GS groups (Figure 4C,D). Among those following a GFD, 58% (n = 54) were seeing a health professional for dietary advice.

## 4. Discussion

In this study, the general prevalence of SR-GS was lower than that reported in other Latin American (3.1% vs. 5.3–7.8%) [9,10,11] and European countries (3.1% vs. 6.2–13%) [7,8]. Certainly, the prevalence of gluten-related disorders, such as CD, could be associated with the consumption of wheat among other environmental factors [19]. According to Central America Data [20], the wheat consumption in El Salvador is about 217.92 thousand tons per month; this accounts for an annual wheat consumption of ≈34.34 Kg per capita. Moreover, the basic market basket in urban zones from this country included 1.47 KG of wheat-based products per capita in 2017 [21]. This wheat consumption is lower than that reported for other Latin American countries, such as Argentina and Mexico [15], and that of the European region [19], and could in part explain the low prevalence rate of SR-GS in El Salvador.

The prevalence rates of adherence to a gluten-free diet and gluten avoidance were significantly higher among those aged ≥39 years old. This is in line with previous survey studies carried out in Mexico, Colombia and the United States [10,11,22], but inconsistent with the results obtained for the Argentinian population [9]. The motivations for following the diet or avoiding wheat/gluten from the diet were assessed in Salvadoran and Argentinian population. In these two studies, the main motivations were: weight control, the symptoms triggered by wheat/gluten intake, and the perception that a gluten-free diet is healthier. This highlights that motivation *per se* is not enough to explain the discrepancies among the age of the gluten-free diet followers or wheat/gluten avoiders among populations. Other factors such, as media attention [7], could be assessed in future studies to better explain such differences.

Self-reported conditions such as IBS, food intolerance, and colitis were significantly associated with recurrent adverse reactions to gluten ingestion. Previous survey studies have documented that these conditions are more common in SR-GS than in non-SR-GS individuals [7,8,9,10,11]. Particularly, more than 40% of those who met Rome III criteria for IBS also met criteria for SR-NCGS [23]. The gastrointestinal symptoms triggered by gluten-containing products in IBS patients could be attributed to the presence of fermentable, oligo-, di-, monosaccharides, and polyols (FODMAPs) in such products [24,25]. Experts in the field of gluten-related disorders have proposed a double-blinded placebo-controlled gluten challenge for the diagnosis of NCGS [6], but gluten challenges are not commonly performed in clinical practice [8]. Overall, our data collected under a survey-based approach support the notion that gluten has a strong association with IBS [9,23].

The prevalence rate of SR-PD celiac disease in Salvadoran population was 0.15%. This is higher than the prevalence rates estimated in Mexicans (0.08%) [11] and Colombians (no CD cases reported) [10], but lower than that observed in Argentinians (0.58%) [9], who reported the expected prevalence rate among populations (between 0.5% and 15%) [26]. Similar to Mexico and Colombia, El Salvador’s ministry of health has no implemented programs for the detection and control of CD. Additional to this, the possibility to educate healthcare personnel regarding gluten-related disorders is an issue that deserves to be explored, as seven physician-diagnosed respondents reported the co-existence of NCGS with CD and/or WA. According to experts on gluten-related disorders, both CD and WA should be ruled out in diagnoses of NCGS [2,3,6]. Therefore, based on current knowledge and scientific consensus, CD or WA does not co-exist with NCGS, but it is not uncommon that some physicians misunderstand the spectrum of gluten-related disorders [27,28]. Certainly, other studies have highlighted that in clinical practice in El Salvador, most cases of CD are confirmed by gastro-endoscopy with duodenal biopsies (biopsy-proven CD) [29].

In this study, the most commonly reported gastrointestinal and extraintestinal symptoms triggered after gluten ingestion were bloating, stomachache, headache, and tiredness. These symptoms are consistent with previous studies carried out in Latin America [9,10,11] and Europe [8,30]. However, these symptoms are not truly representative of recurrent adverse reactions to gluten, as they are also frequently reported by people complaining of adverse reactions to the ingestions of foods other than gluten [11].

Regarding the prevalence of adherence to a GFD, 7% of the studied population reported to be following a GFD. This prevalence rate is higher than that reported in Argentinians (6.37%), which was considered in 2017 to have the highest self-reported prevalence rate of adherence to a GFD ever observed [9]. Similar to previous studies [9], most SR-GS individuals reported that they were following a GFD or avoiding wheat/gluten from their diets due to symptomatic relapse. Notably, most SR-GS individuals (84%) reported that dietitians and general practitioners were instructing them about the GFD. Although being instructed about diet by a health professional does not guarantee that the individual will overcome the impact that GFD has on his/her quality of life [31], appropriate gluten-free dietary counseling and explanation about the reasons for following a GFD could be helpful to increase awareness about diet and, and to avoid long-term complications in some cases. Most non-SR-GS individuals were following a GFD for reasons such as weight control, and because of a notion that a GFD is healthier, consistent with previous studies [32,33]. Among the SR-GS individuals, the main reasons for gluten-free dietary non-compliance were the mildness of the symptoms triggered after wheat/gluten intake and/or the low availability and high cost of gluten-free products, as we and others stated previously [7,9].

The main limitation of this study is that it was self-reported, and our data were not confirmed by objective diagnostic criteria such as skin prick tests, specific IgEs, celiac serology or HLA typing, or oral gluten challenges. The major strengths are its population-based design, the large number of respondents, and the criteria used to estimate immediate-type food allergy. It has been shown that up to 93% of subjects fulfilling these criteria had IgE antibodies to the implicated food [34]. Overall, this study provides useful epidemiological data regarding gluten-related disorders and adherence to a GFD in Salvadoran population, and serves as groundwork for further studies based on objective diagnostic criteria.

## 5. Conclusions

The results highlight that both recurrent-adverse reactions to wheat/gluten, and the physician-diagnosis of gluten-related disorders, are common in Salvadoran population. However, a physician diagnosis of NCGS, along with CD and/or WA, was also commonly reported. The prevalence of adherence to a GFD in Salvadoran population is the highest ever reported, but most people following a GFD were doing it for reasons other than health-related benefits, and almost half of these without medical/dietitian advice. To our knowledge, this is the first population-based study conducted in Central America to evaluate the prevalence of gluten-related disorders and adherence to a GFD. We should acknowledge that the main use of our study is to serve as the groundwork for future epidemiological studies based on objective diagnostic criteria, such as wheat/gluten oral challenges for the diagnosis of WA or NCGS, or celiac serology, followed by gastro-endoscopy with duodenal biopsies for the diagnosis of CD.

## Figures and Tables

**Table 1:** Definitions utilized in this study.

Condition		Definition
Adverse reaction to food		Subjects who reported that the food-induced symptoms occurred always or most of the time (recurrent) or sometimes (non-recurrent).
Self-reported physician-diagnosed (SR-PD *) celiac disease (CD *)		Subjects who reported that a physician or a health professional diagnosed celiac disease and were also following a GFD *.
Wheat allergy	SR-PD wheat allergy (WA *)	Subjects who reported that a physician diagnosed wheat allergy and were also following a GFD *
	SR-WA *	Subjects who reported recurrent adverse reactions convincing of food allergy.
SR-GS *		Subjects who met criteria for recurrent adverse reactions to oral wheat/gluten and were also following a GFD *.
NCGS **	SR-PD NCGS *	Subjects who reported that a physician diagnosed them and were also following a GFD *.
	SR-NCGS *	Subjects who met the following: (1) individuals who did not meet criteria for self-reported physician-diagnosed CD * or wheat allergy; (2) individuals who did not meet criteria for self-reported wheat allergy; and (3) individuals who met criteria for SR-GS *.

* Acronyms used: SR-PD: Self-reported Physician-Diagnosed; CD: Celiac Disease; GDF: Gluten-Free Diet; WA: Wheat Allergy; SR-WA: Self-Reported Wheat Allergy; SR-GS: Self-Reported Gluten-Sensitivity; NCGS: Non-Celiac Gluten Sensitivity; SR-NCGS; Self-Reported Non-Celiac Gluten Sensitivity. ** NCGS cases reported to co-exist with WA and/or CD were excluded for prevalence estimations or classified according to the symptoms reported.

**Table 2:** Self-reported prevalence rates.

Condition	(+) Cases *	Mean Age in Years (range)	Prevalence by Gender (95% CI)	*p* Value	General Prevalence (95% CI)
Adverse reaction to foods	Total = 272	37.14 (18–84)	M 9.7 (8.2–11.4) F 10.8 (9.2–12.6)	0.222	20.5 (18.5–22.85)
	M = 129				
	F = 144				
Adverse reaction to wheat/gluten	Total = 60	42.23 (19–77)	M 1.8 (1.2–2.6) F 2.7 (1.9–3.7)	0.112	4.5 (3.5–5.7)
	M = 24				
	F = 36				
(a) Self-Reported Gluten sensitivity (SR-GS **)	Total = 41	43.65 (20–72)	M 0.9 (0.5–1.6) F 2.1(1.4–3.0)	0.011	3.1 (2.3–4.2)
	M = 13				
	F = 28				
(b) SR-PD ** Celiac disease	Total = 2	43.5 (26–61)	M 0.07(0.003–0.3) F 0.07(0.003–0.3)	1.000	0.15 (0.04–0.5)
	M = 1				
	F = 1				
(c) Wheat allergy	Total = 10	48.1 (30–72)	M 0.3 (0.1–0.7) F 0.4 (0.2–0.9)	0.542	0.75 (0.4–1.3)
	M = 4				
	F = 6				
(d) NCGS **	Total = 13	43.69(27–55)	M 0.3 (0.1–0.7) F 0.6 (0.3–1.2)	0.172	0.98 (0.5–1.6)
	M = 4				
	F = 9				
Adherence to GFD **	Total = 93	41.75 (18–72)	M 3.5 (2.6–4.6) F 3.4 (2.6–4.5)	1.000	7.0 (5.7–8.5)
	M = 47				
	F = 46				
Avoid wheat/gluten-containing foods	Total = 110	42.0 (18–77)	M 3.4 (2.5–4.5) F 4.9 (3.8–6.2)	0.036	8.3 (6.9–9.9)
	M = 45				
	F = 65				

* Positive cases, ** Acronyms used: SR-GS: Self-Reported Gluten Sensitivity; SR-PD: Self-Reported Physician-Diagnosed; NCGS: Non-Celiac Gluten Sensitivity; GFD: Gluten-Free Diet.

**Table 3:** Individuals who reported the coexistence of NCGS with other gluten-related disorders.

Individual ID	Diagnosed by	Diagnosed Disorders	Following a GFD *	Excluded or Re-Classified	Criteria for Exclusion/Inclusion
0042	Homeopathic physician	WA * CD * NCGS *	Yes	Excluded	CD or WA does not co-exist with NCGS. The self-reported symptoms did not met criteria for WA
0331	Pediatrician	WA * NCGS *	Yes	Re-classified	The self-reported symptoms were convincing of WA
0550	Physician				
0602	Allergologist				
0742	Dietitian	WA * NCGS *	Yes	Excluded	CD or WA does not co-exist with NCGS. The self-reported symptoms did not met criteria for WA
0743	Physician	WA * CD * NCGS *	No		
1314	Gastroenterologist	WA * CD * NCGS *	Yes		

* Acronyms used: GDF: Gluten-Free Diet; CD: Celiac Disease; WA: Wheat Allergy; NCGS: Non Celiac Gluten Sensitivity.
